# Real-Time Dissemination of Aggregate Data on Presentation and Outcomes of Patients With Venous Thromboembolism: The RIETE Infographics Project

**DOI:** 10.1177/1076029620931200

**Published:** 2020-09-16

**Authors:** Alfonso Tafur, Behnood Bikdeli, Ido Weinberg, David Jimenez, Annia Monreal, Raquel Barba, Enrique Mira, Victor Macrinici, Harlan M. Krumholz, Mayra Hawkins, Manuel Monreal, the RIETE Investigators

**Affiliations:** 1 Medicine, Vascular Medicine, North Shore University Health System, Evanston, IL, USA; 2 University of Chicago, Pritzker School of Medicine, IL, USA; 3 NewYork-Presbyterian Hospital/Columbia University Irving Medical Center, New York, USA; 4 Clinical Trials Center, Cardiovascular Research Foundation, New York, USA; 5 Center for Outcomes Research and Evaluation (CORE), Yale School of Medicine, Connecticut, USA; 6 Vascular Medicine, Division of Cardiology, Fireman Vascular Center, Massachusetts General Hospital, Boston, MA, USA; 7 Department of Respiratory, Hospital Ramon y Cajal, Universidad de Alcala (IRYCIS), Madrid, Spain; 8 RIETE Registry Coordinating Center, S&H Medical Science Service, Madrid, Spain; 9 Department of Internal Medicine, Hospital Universitario Rey Juan Carlos, Madrid, Spain; 10 Department of Internal Medicine, Hospital Universitario Virgen de la Arrixaca, Murcia, Spain; 11 Department of Internal Medicine, Hospital Germans Trias i Pujol, Universidad Autonoma de Barcelona, Badalona, Barcelona, Spain

**Keywords:** thrombosis, pulmonary embolism, risk assessment

## Abstract

In the current era of patient empowerment and precision medicine, access to timely information is critical to decision-making. Unfortunately, we currently lack patient-specific, real-time data about clinical presentation, risk of thrombotic or hemorrhagic events, key risk factors, and adverse outcomes in patients with venous thromboembolism (VTE). Accordingly, the Registro Informatizado Enfermedad TromboEmbólica (RIETE) investigators developed a tool to provide an open-source, real-time graphic representation of VTE-related data derived from over 90 000 patients with confirmed VTE. This information is intended to facilitate discussion in the informed decision-making process. The current article describes the aims, rationale, methods, and ongoing and future efforts of the real-time VTE infographics developed by the RIETE registry collaborators.

## Introduction

Venous thromboembolism (VTE), including deep vein thrombosis (DVT) and pulmonary embolism, is a common, dangerous, and, often, preventable cause of death and disability.^1,2^ The management of patients with VTE is complex, with a myriad of options for short- and long-term treatment. However, we lack timely data about clinical presentation, risk of thrombotic or hemorrhagic events, and key risk factors or adverse outcomes in patients with VTE. Estimates of risk (of both recurrence and treatment-related adverse events) are important to decision-making about preventive, diagnostic, and therapeutic strategies.

The Registro Informatizado Enfermedad TromboEmbólica (RIETE) registry is ideally positioned to provide information about VTE. This is a global registry with over 200 participating centers in 20 countries. At the time of writing this manuscript, the registry includes data from over 90 000 patients. This registry is poised to offer real-time patient-centric information that can be constantly updated. Thus, the RIETE investigators have developed an infographics platform for the purpose of real-time data analysis and information sharing intended both for patients and for health care professionals. Herein, we describe the methodology, advantages, and limitations for the design and use of this infographics platform.

## Methods

### Overview

The RIETE is a large prospective multinational ongoing registry of consecutive patients with objectively confirmed VTE (ClinicalTrials.gov identifier: NCT02832245). The RIETE has helped characterize the risk factors, pattern of presentation, and clinical outcomes of patients with VTE, including in the aforementioned understudied subgroups: cancer, renal insufficiency, liver cirrhosis, recent major bleeding, very elderly patients, or pregnancy.^3–8^ The RIETE has recently expanded to collect long-term outcomes data and has broadened its inclusion criteria to enroll other forms of venous thrombosis (such as cerebral, splanchnic, superficial, or retinal vein thrombosis). The RIETE platform is also being used to conduct pragmatic comparative effectiveness studies, including randomized controlled trials (RCTs).

### Goals of the Real-Time Infographics Software

Our overarching goal is to provide real-time information collected during real-life VTE care in a format designed to help patients, health care providers, scholars, and policymakers with relevant and timely information from a large ongoing prospective multinational registry. We hope our platform will become an instrument to facilitate shared decision-making, especially for patients with characteristics underrepresented in clinical trials.

### Platform Development

The website *www.trombo.info* allows patients, clinicians, and/or investigators to communicate their concerns or questions. The website has over 1.5 million visits per year. Classification of the first 1500 questions submitted from April 2015 to December 2018 by RIETE investigators (MM, AM, DJ, MH, and others) led to selection of the most frequent queries: (1) How often do symptoms persist after a DVT? (48% of questions); (2) What could I do to prevent a VTE? (30%); and (3) What is the risk for recurrences? (12%).

Additionally, questions arising from the RIETE Network of 200 active institutions globally served as guide for clinical scenario development. Thus, search for occult cancer after a VTE event was a selected query. We have formed a RIETE Real-Time Infographics steering committee (AT, BB, IW, and MM) to organize prospective query selection and dissemination strategy.

The RIETE data are queried for quality control every week, and the real-time analysis platform is updated on a monthly basis. Infographics already available include queries on VTE risk factors, cancer-associated thrombosis, occult cancer probability, post thrombotic syndrome, and so on. At present, the infographics are optimized to color coding of frequency data. The platform is built to be interactive and responds to multiple options of query personalization. Current and expected capabilities of the platform are summarized in Table 1. All enrolled patients provide written or verbal informed consent according to the local ethics protocols.

**Table 1.** Present Aspects and Future Directions of the RIETE Infographics Project.

	Present	Future
Involving, informing, and receiving feedback from patients	Yes	Yes
Involving, informing, and receiving feedback from RIETE investigators	Yes	Yes
Involving, informing, and receiving feedback from non-RIETE investigators	Yes	Yes
Involving, informing, and receiving feedback from policymakers	No	Yes
Conducting research based on the infographics platform	No	Yes
Designing quality improvement initiatives based on the infographics platform	No	Yes

Abbreviation: RIETE, Registro Informatizado Enfermedad TromboEmbólica.

### Definitions

A comprehensive list of the main methods and definitions in RIETE has been published.^9^ The main outcomes presented in the infographics are initially limited to all-cause mortality, recurrent VTE, major bleeding, and clinically relevant nonmajor bleeding. Data elements are collected for a minimum of 3 months, and there is ongoing centralized quality control of the data.

### Examples

Currently, there are 6 interactive infographics available. The following are examples:

### Risk Factors for VTE

**Figure 1.**
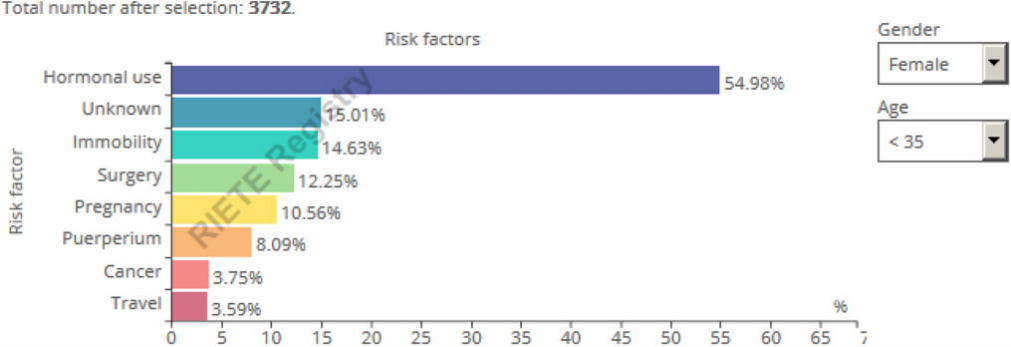
Real-time risk factor Registro Informatizado Enfermedad TromboEmbólica (RIETE) infographic. Query October 2019.

Accurate identification of at-risk patients may help to increase awareness about early signs and symptoms of VTE. Therefore, and also in response to patient queries, a real-time infographic that presents the frequency data of conventional risk factors stratified by age or gender was created. For instance, in RIETE, 50% of men but only 40% of women had unprovoked VTE (Figure 1). Hence, there is potential to prevent 50% of VTE in men and 60% of VTE in women with improved prophylaxis. When applying the filter for only women aged <35 years, we show that over 50% of them were using hormonal therapy, 10% were pregnant, and 8% were postpartum, while in 15% there was no known risk factor (Figure 1).

#### Venous thromboembolism after surgery

**Figure 2.**
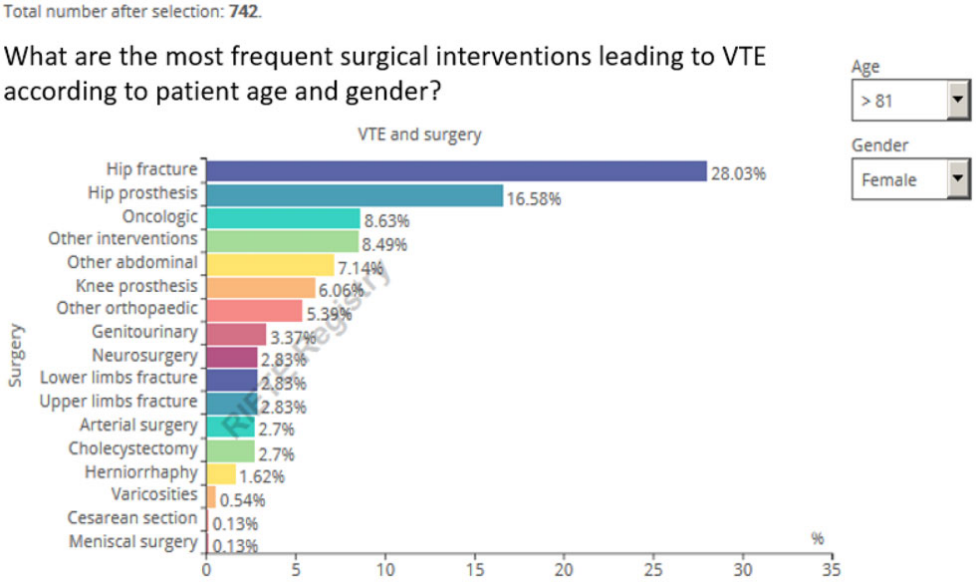
Real-time Registro Informatizado Enfermedad TromboEmbólica (RIETE) infographic on surgery type as VTE risk factor. Query October 2019.

The following figure illustrates the most common surgical interventions for the whole cohort of patients. As with all the infographics, users can filter the data (Figure 2). For instance, among 742 women aged >85 years, hip or knee surgery (either elective or for hip fracture) accounted for over half of the interventions. Among 501 women aged <35 years, cesarean delivery accounted for 23.8% of the most frequent interventions leading to VTE, followed by genitourinary surgery (17.4%).

### Ethics and Future Research

We realize that forward-looking presentations of the aggregate data may influence decision-making. Thus, we are constructing a parallel study to quantify the effectiveness of this platform as a patient education tool. Also, we plan to test the possibility of affecting physician behavior and their degree of confidence in practicing according to published standards of care after being exposed to data that represents a more narrow demographic (ie, more similar to their patients), when compared to RCTs. Moreover, acknowledging that the visualized data might affect patient–physician behavior while also respecting freedom of choice,^10,11^ we will not generate specific therapy recommendations, and the platform will remain as an informative tool rather than an alternative to medical advice.

## Discussion

As we have demonstrated, a free to access, constantly updated, real-life platform has been created based on an international registry of over 90 000 patients to offer some insight into the products of these data. This information is not meant to be used as medical advice nor to change the management recommendations as defined by international guidelines. Randomized trials and sophisticated observational comparative effectiveness studies continue to have their important roles. Certainly, at the current stage of development, the infographics are not a platform to conduct comparative effectiveness studies or to make real-time decision about comparative effectiveness or safety of treatment alternatives. However, we speculate that this tool may potentially impact health literacy and will help prioritize future research questions and VTE-care policy.

Advocacy for shared decision-making is gaining momentum in health care policy.^12^ Yet, there is a paucity of validated tools to facilitate VTE-specific questions. In contrast, there is high-level evidence that decision aids with clarification exercises will improve value-based patient choices and may also improve the patient–practitioner communication.^13,14^ Indeed, patients with VTE recognize and dislike the lack of facilitated communication to establish expectations and management choices.^15^ In its current form, the RIETE infographic project is predominantly informative (Table 1).

Less is known about quantification of the extent to which decision aids are needed to have a positive effect. Thus, in order to validate our tool, we propose a multistep project (Table 2) anchored on the readily available real-time infographics. In the first step, we will optimize the framework of presentation of the infographic data using patient and physician feedback measured within a qualitative study. With information anchored on focused groups, and in consultation with behavioral scientists and a marketing specialist, we will generate an infographic optimized for a shared decision-making process. In phase 2 of our research, we will test the hypothesis that the format of data presentation to the physician may alter the degree of confidence in, and perception of, VTE management priorities. To this end, we will test data presentation options to physicians in the RIETE network and cross validate the findings outside of RIETE. A primary strength of the RIETE network is that we will be able to test our assumptions in multiple countries and cultural backgrounds. To stress transparency of the data, the infographic webpage clarifies the date of the aggregate and patients included, which are rigorously submitted to the same quality control as the rest of the RIETE registry.^9^ The presentation and development of the second-generation RIETE real-time infographics will be designed within the framework of libertarian paternalism, acknowledging that the visualized data might affect patient–physician behavior while also respecting freedom of choice.^10,11^ We will hone our efforts to expose existing gaps of knowledge where there is paucity of RCT data and clearly defined guideline directives. Finally, the third segment of our research will measure patient satisfaction scores in a cluster samples RCT using the RIETE Network, in which the intervention will be the utilization of our optimized infographic platform as a tool to facilitate the shared decision-making process.

**Table 2.** The RIETE Real-Time VTE Infographic Shared Decision-Making Validation.

Phase 1. Framework optimization Qualitative research – Focus group interviews of patients and relatives of patients with history of VTE. Query preferences of information facilitation formats and hierarchy of content. – Focus group of physicians. Query perceived areas of higher communication uncertainty with the patients. – Discourse analysis of focus groups. Generate visually optimized real-time infographics in response to patient–physician communication needs
Phase 2. Confidence measure Quantitative, survey research – Query RIETE network participants. We will present in a case format, common, or underrepresented patient populations in VTE literature and measure the degree of confidence in management. Then, we will requery management confidence after presentation of RIETE-generated scenario-specific infographic.
Phase 3. Testing Quantitative, randomized controlled trial – To measure patient-satisfaction among patients with VTE diagnosis. The intervention will be real-time infographic-facilitated shared decision-making using RIETE data.

Abbreviation: RIETE, Registro Informatizado Enfermedad TromboEmbólica; VTE, venous thromboembolism.

In conclusion, the current article provides a summary of an ongoing effort to develop an infographics platform to share relevant information about demographics, presentation, and outcomes of patients with VTE. Shared decision-making needs to harmonize evidence-based medicine, physician experience, and patient preference. These constantly updated, free to access, data could be informative for patients, clinicians, and policy makers and may help in the generation of better knowledge to help improve patient care. The proposed platform is aimed to help preserve patient autonomy while facilitating educated medical choices. Interested researchers are invited to join the RIETE investigators.

## Appendix A

*Coordinator of the RIETE Registry:* Manuel Monreal.

*RIETE Steering Committee Members:* Paolo Prandoni, Benjamin Brenner and Dominique Farge-Bancel.

*RIETE National Coordinators:* Raquel Barba (Spain), Pierpaolo Di Micco (Italy), Laurent Bertoletti (France), Sebastian Schellong (Germany), Inna Tzoran (Israel), Abilio Reis (Portugal), Marijan Bosevski (R. Macedonia), Henri Bounameaux (Switzerland), Radovan Malý (Czech Republic), Peter Verhamme (Belgium), Joseph A. Caprini (USA), Hanh My Bui (Vietnam).

*RIETE Registry Coordinating Center:* S & H Medical Science Service.

Members of the RIETE Group

Spain: Adarraga MD, Agud M, Aibar J, Aibar MA, Alfonso J, Amado C, Arcelus JI, Baeza C, Ballaz A, Barba R, Barbagelata C, Barrón M, Barrón-Andrés B, Blanco-Molina A, Camon AM, Cañas I, Castro J, Cerdà P, Criado J, de Ancos C, de Miguel J, del Toro J, Demelo-Rodríguez P, Díaz-Peromingo JA, Díez-Sierra J, Díaz-Simón R, Domínguez IM, Encabo M, Escribano JC, Falgá C, Farfán AI, Fernández-Capitán C, Fernández-Reyes JL, Fidalgo MA, Flores K, Font C, Font L, Francisco I, Gabara C, Galeano-Valle F, García MA, García-Bragado F, García-Mullor MM, Gavín-Blanco O, Gavín-Sebastián O, Gil-Díaz A, Gómez-Cuervo C, González-Martínez J, Grau E, Gutiérrez J, Hernández-Blasco L, Jara-Palomares L, Jaras MJ, Jiménez D, Joya MD, Jou I, Lacruz B, Lecumberri R, Lima J, Lobo JL, López-Brull H, López-Jiménez L, López-Miguel P, López-Núñez JJ, López-Reyes R, López-Sáez JB, Lorente MA, Lorenzo A, Loring M, Madridano O, Maestre A, Marchena PJ, Martín del Pozo M, Martín-Guerra JM, Martín-Martos F, Mella C, Mellado M, Mercado MI, Moisés J, Monreal M, Morales MV, Muñoz-Blanco A, Muñoz-Guglielmetti D, Nieto JA, Núñez MJ, Olivares MC, Ortega-Michel C, Ortega-Recio MD, Osorio J, Otero R, Parra P, Parra V, Pedrajas JM, Pellejero G, Pérez-Jacoiste A, Peris ML, Pesántez D, Porras JA, Ramos E, Reig L, Riera-Mestre A, Rivas A, Rodríguez-Cobo A, Rodríguez-Matute C, Rosa V, Rubio CM, Ruiz-Artacho P, Ruiz-Giménez N, Ruiz-Ruiz J, Ruiz-Sada P, Sahuquillo JC, Salgueiro G, Sampériz A, Sánchez-Muñoz-Torrero JF, Sancho T, Soler S, Suárez S, Suriñach JM, Tiberio G, Torres MI, Tolosa C, Trujillo-Santos J, Uresandi F, Usandizaga E, Valle R, Vela JR, Vidal G, Villares P, Zamora C; Argentina: Gutiérrez P, Vázquez FJ; Belgium: Vanassche T, Vandenbriele C, Verhamme P; Czech Republic: Hirmerova J, Malý R; Ecuador: Salgado E; France: Benzidia I, Bertoletti L, Bura-Riviere A, Crichi B, Debourdeau P, Espitia O, Farge-Bancel D, Helfer H, Mahé I, Moustafa F, Poenou G; Germany: Schellong S; Israel: Braester A, Brenner B, Tzoran I; Italy: Amitrano M, Bilora F, Bortoluzzi C, Brandolin B, Bucherini E, Ciammaichella M, Colaizzo D, Dentali F, Di Micco P, Giammarino E, Grandone E, Mangiacapra S, Mastroiacovo D, Maida R, Mumoli N, Pace F, Pesavento R, Pomero F, Prandoni P, Quintavalla R, Rocci A, Siniscalchi C, Tufano A, Visonà A, Vo Hong N, Zalunardo B; Latvia: Gibietis V, Kigitovica D, Skride A; Portugal: Ferreira M, Fonseca S, Martins F, Meireles J; Republic of Macedonia: Bosevski M, Zdraveska M; Switzerland: Bounameaux H, Mazzolai L; USA: Bikdeli B, Caprini JA, Tafur AJ, Weinberg I, Wilkins H; Vietnam: Bui HM.
